# Supplementary material for: Engineering fungal de novo fatty acid synthesis for short chain fatty acid production
Source: Nat Commun. 2017 Mar 10;8:14650. doi: 10.1038/ncomms14650 (PMC5353594; doi:10.1038/ncomms14650)
Supplement: Supplementary Information — Supplementary Figures and Supplementary Tables [file ncomms14650-s1.pdf]

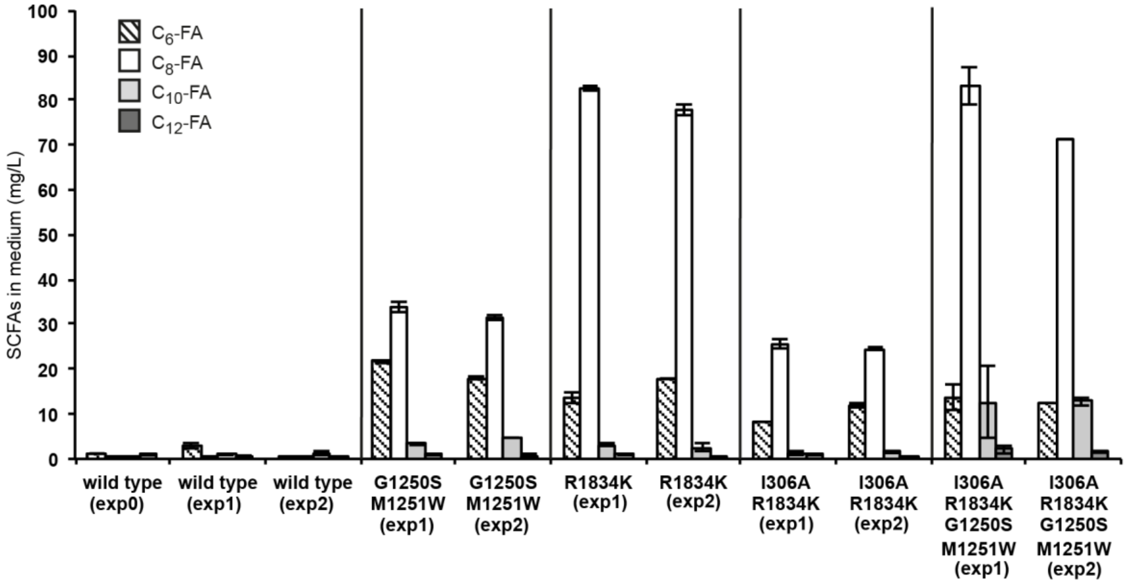

Supplementary Figure 1: Product spectra of FAS engineered strains in YPD medium. For data collection, cultures of *S. cerevisiae* strains were grown for 48 h at 30 °C, the media extracted and FA quantified via GC-FID (for more information see Online Methods). Error bars reflect the standard deviation from three technical replicates. The figure compares two or three biological replicates, and demonstrates the high repeatability of data. Data labeled as experiment 1 (exp1) are shown in Fig. 2B (see also Supplementary Table 1).

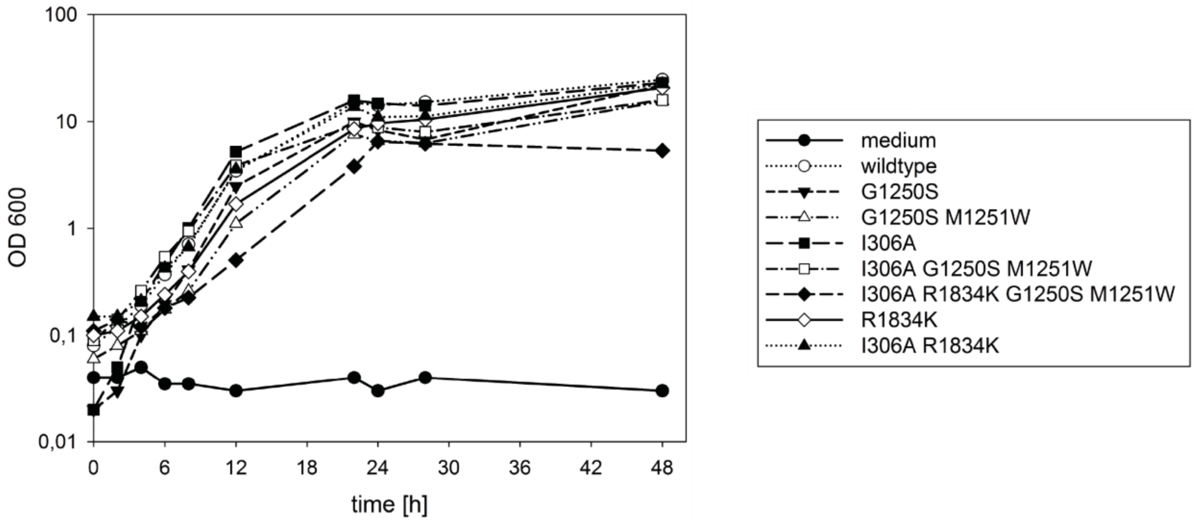

Supplementary Figure 2: Growth properties of FAS engineered strains. Growth curves of engineered yeast strains**.** For selected strains, the cell density was monitored at several time points (average of two measurements from one culture).

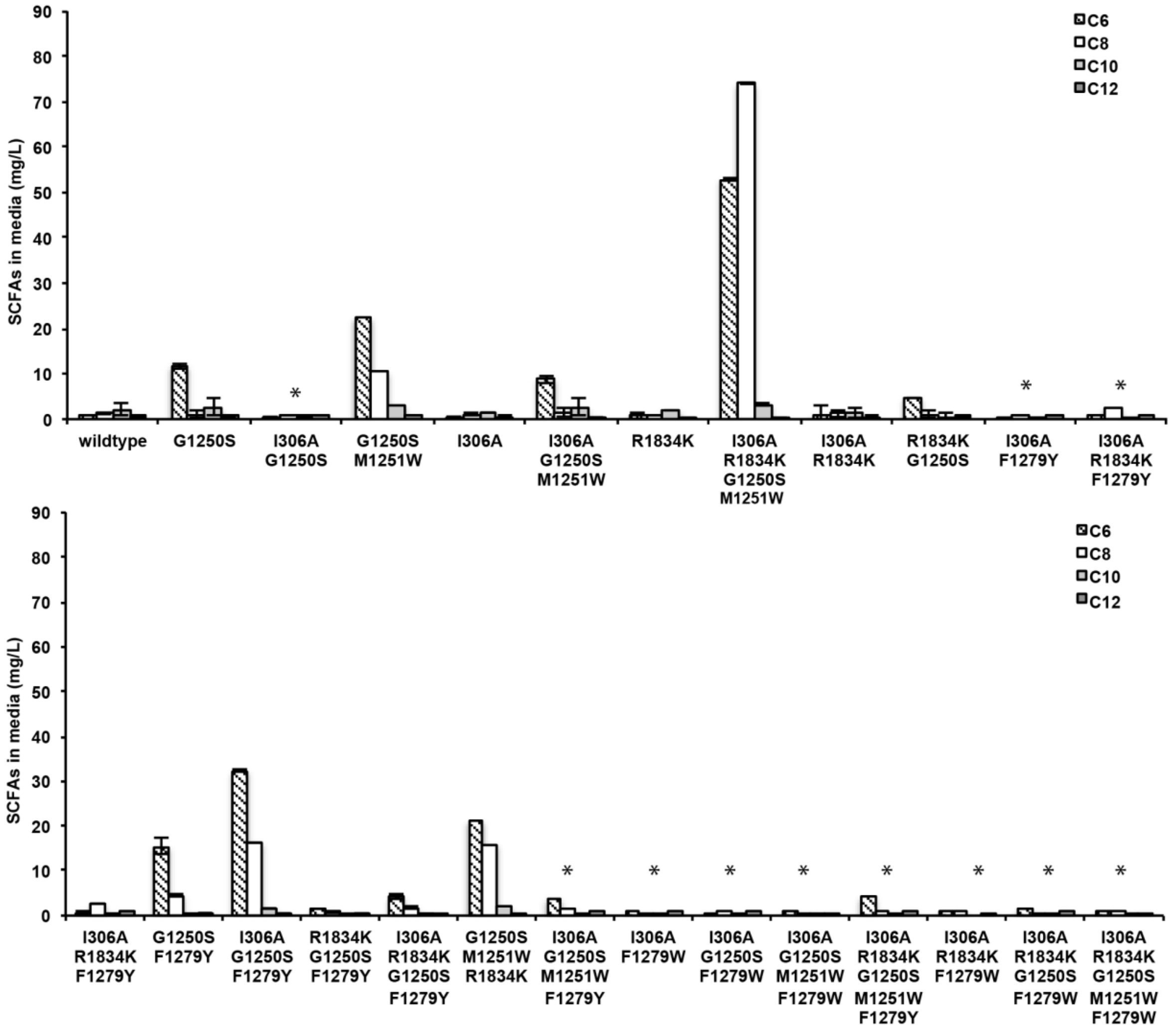

Supplementary Figure 3: Product spectra of FAS engineered strains in YPD medium complemented with oleic acid. All strains were grown in YPD supplemented with $C_{18:1}$ (1 mM). Error bars reflect the standard deviation from three technical replicates (see Fig. 2B), except that measurements marked with (*), which showed low concentrations in an initial screening, have not been repeated.

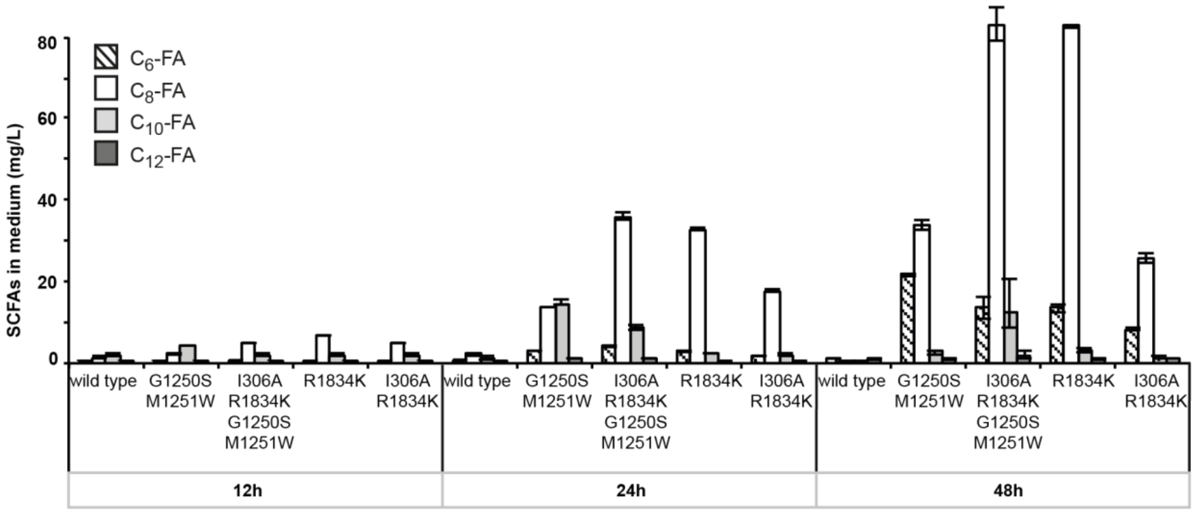

Supplementary Figure 4: FA production during culture growth. Product spectra of selected strains recorded at various time points during growth. Besides the regular product spectra after 48 h, additional measurements were performed for selected strains after 12 h and 24 h. Data were recorded under growth conditions and with statistics as described in Fig. 2B.

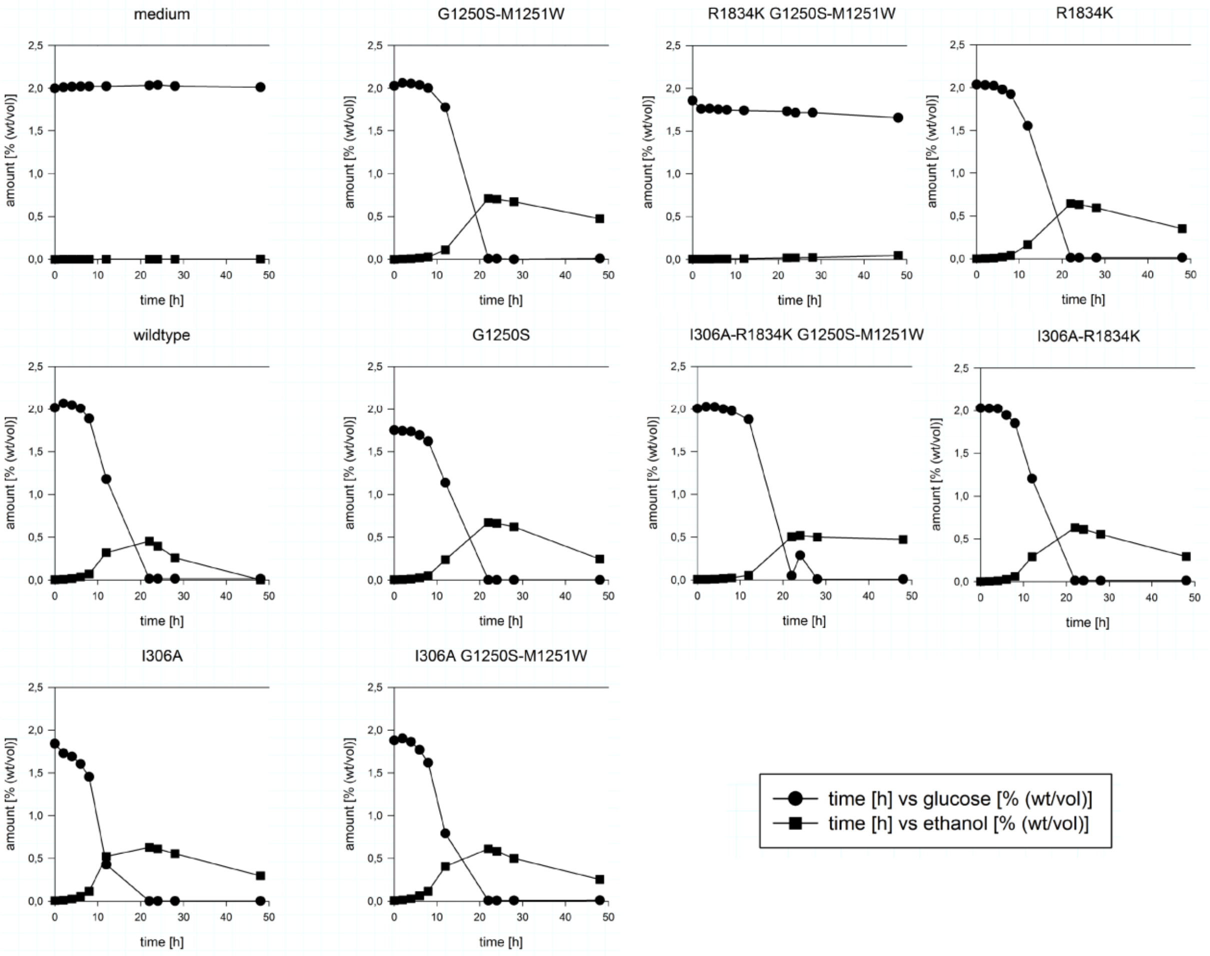

Supplementary Figure 5: Glucose and ethanol concentrations during yeast cultivations. For selected strains, the supernatants of the medium were monitored at several time points during the 48 h cultivation. The amount of remaining glucose and produced ethanol in the fermentation medium was measured by HPLC. The shown data are results from one typical experiment.

Supplementary Table 1: Titers of SCFA detected in media. Tables present data of two series of independent experiments (independent biological samples), termed “experiment 1” and “experiment 2”. The Summary Table shows mean of two biological samples (n = 2), as well as data on other mutants collected from one biological sample each. Each value has been determined in triplicate. Please note that values from the Experiment 1 have been used for Fig. 2B, and values from Summary have been used, when SCFA titers are described in the text.

Experiment 1		C6	C8	C10	C12	C14
	**wildtype**	2.83	0.53	0.97	0.78	0.73
	**G1250S**	20.59	4.27	1.33	0.76	0.51
	**G1250S M1251W**	21.79	33.81	3.23	0.99	1.27
	**I306A**	1.89	0.47	1.16	0.68	0.97
	**G1250S M1251W I306A**	36.41	28.63	2.32	0.91	0.79
	**G1250S M1251W R1834K**	1.23	0.61	1.79	1.38	1.76
	**G1250S M1251W R1834K I306A**	13.63	83.03	12.64	2.05	2.08
	**R1834K**	13.48	82.66	3.21	1.04	1.49
	**R1834K I306A**	8.37	25.74	1.22	1.07	1.12

Experiment 2		C6	C8	C10	C12	C14
	**wildtype**	0.54	0.64	1.31	0.42	0.06
	**G1250S**	9.93	3.59	2.34	0.19	0.10
	**G1250S M1251W**	17.94	31.60	4.83	0.80	0.64
	**I306A**	0.51	0.45	1.19	0.27	0.05
	**G1250S M1251W I306A**	37.87	22.48	2.91	0.40	0.08
	**G1250S M1251W R1834K**	2.20	3.23	1.90	0.44	0.08
	**G1250S M1251W R1834K I306A**	12.42	71.17	12.84	1.50	0.22
	**R1834K**	18.00	77.75	2.47	0.45	0.10
	**R1834K I306A**	11.85	24.52	1.66	0.47	0.11

Summary		C6	C8	C10	C12	C14	n
	**wildtype**	1.68	0.58	1.14	0.88	0.58	2
	**G1250S**	15.26	3.93	1.83	0.59	0.35	2
	**G1250S M1251W**	19.87	32.71	4.03	0.59	1.03	2
	**I306A**	1.20	0.46	1.18	0.74	0.62	2
	**G1250S M1251W I306A**	37.14	25.56	2.61	0.59	0.60	2
	**G1250S M1251W R1834K**	1.71	1.92	1.84	0.89	1.10	2
	**G1250S M1251W R1834K I306A**	13.03	77.10	12.74	1.24	1.79	2
	**R1834K**	15.74	80.20	2.84	1.27	0.97	2
	**R1834K I306A**	10.11	25.13	1.44	0.76	0.80	2
	**I306A G1250S**	20.41	1.01	0.37	1.01		1
	**R1834K G1250S**	9.38	26.70	2.51	0.88		1
	**I306A R1834K G1250S**	52.12	63.07	2.19	0.80		1
	**G1250S F1279Y**	0.81	0.75	0.77	0.79		1
	**I306A G1250S F1279Y**	1.35	1.56	2.61	0.79		1
	**R1834K G1250S F1279Y**	0.65	0.29	0.22	0.94		1
	**I306A R1834K G1250S F1279Y**	0.62	0.28	0.18	0.99		1
	**I306A F1279Y**	14.72	5.50	0.25	0.32		1
	**I306A R1834K F1279Y**	2.20	47.87	3.24	0.63		1

Supplementary Table 2: Change of FA levels after introduction of FAS mutations. For the quantification of the specific impact of a mutation, average yields of the strain without the respective mutation are compared to average yields of the strain carrying the mutation (second line). The ratio of values (second value divided by the first value) is given as a factor, specifying the x-fold increase of the mutation to the yield (C_6, C_8 and total short FA). Accordingly, values above 1 (in bold) are reporting a higher yield, and values below 1 a lower yield caused by the mutation. For the data use in this Table, see Supplementary Table 1.

		C6 (mg/L)	C8 (mg/L)	total (C6 to C12) (mg/L)
**G1250S (KS domain)**	wild type	1,7	0,6	4,3
	G1250S	15,3	3,9	21,6
	X-fold increase	**9,1**	**6,7**	**5,0**
	I306A	1,2	0,5	3,6
	I306A G1250S	20,4	1,0	22,8
	X-fold increase	**17,0**	**2,2**	**6,4**
	R1834K	15,7	80,2	100,1
	R1834K G1250S	9,4	26,7	39,5
	X-fold increase	0,6	0,3	0,4
	I306A R1834K	10,1	25,1	37,4
	I306A R1834K G1250S	52,1	63,1	118,2
	X-fold increase	**5,2**	**2,5**	**3,2**
**M1251W (KS domain)**	G1250S	15,3	3,9	21,6
	G1250S M1251W	19,9	32,7	57,2
	X-fold increase	**1,3**	**8,3**	**2,6**
	I306A G1250S	20,4	1,0	22,8
	I306A G1250S M1251W	37,1	25,6	65,9
	X-fold increase	**1,8**	**25,2**	**2,9**
	I306A R1834K G1250S	52,1	63,1	118,2
	I306A R1834K G1250S M1251W	13,0	77,1	104,1
	X-fold increase	0,2	**1,2**	0,9
**F1279Y (KS domain)**	I306A	1,2	0,5	3,6
	I306A F1279Y	14,7	5,5	20,8
	X-fold increase	**12,3**	**11,9**	**5,8**
	I306A R1834K	10,1	25,1	37,4
	I306A R1834K F1279Y	2,2	47,9	53,9
	X-fold increase	0,2	**1,9**	**1,4**

Supplementary Table S2: (continued)

		C6 mg/L)	C8 (mg/L)	total (C6 to C12) (mg/L)
**I306A (AT domain)**	wild type	1,7	0,6	4,3
	I306A	1,2	0,5	3,6
	X-fold increase	0,7	0,8	0,8
	G1250S	15,3	3,9	21,6
	G1250S I306A	20,4	1,0	22,8
	X-fold increase	**1,3**	0,3	**1,1**
	G1250S M1251W	19,9	32,7	57,2
	G1250S M1251W I306A	37,1	25,6	65,9
	X-fold increase	**1,9**	0,8	**1,2**
	R1834K	15,7	80,2	100,1
	R1834K I306A	10,1	25,1	37,4
	X-fold increase	0,6	0,3	0,4
	R1834K G1250S	9,4	26,7	39,5
	R1834K G1250S I306A	52,1	63,1	118,2
	X-fold increase	**5,6**	**2,4**	**3,0**
**R1834K (MPT domain)**	wild type	1,7	0,6	4,3
	R1834K	15,7	80,2	100,1
	X-fold increase	**9,4**	**137,3**	**23,4**
	G1250S	15,3	3,9	21,6
	G1250S R1834K	9,4	26,7	39,5
	X-fold increase	0,6	**6,8**	**1,8**
	I306A G1250S	20,4	1,0	22,8
	I306A G1250S R1834K	52,1	63,1	118,2
	X-fold increase	**2,6**	**62,2**	**5,2**
	I306A	1,2	0,5	3,6
	I306A R1834K	10,1	25,1	37,4
	X-fold increase	**8,4**	**54,4**	**10,5**
	G1250S M1251W I306A	37,1	25,6	65,9
	G1250S M1251W I306A R1834K	13,0	77,1	104,1
	X-fold increase	0,4	**3,0**	**1,6**

Supplementary Table 3: Cell densities (OD_{600}) and wet pellet weight of *S. cerevisiae* strains. OD_{600} and wet pellet weight (50 ml culture) were recorded for strain grown in regular YPD and in YPD supplemented with $C_{18:1}$ (1 mM). For growth in regular YPD, strains divide into three groups; showing (i) regular growth (white background), (ii) reduced growth (light gray) and (iii) very little/no growth (dark gray). In YPD supplemented with $C_{18:1}$, all samples showed essentially the same densities. For strains marked with (*), no significant growth was observed. Values are given for one typical culture. OD_{600} values are given as an average of two measurements from one culture.

	Samples in regular YPD		Samples in YPD with $C_{18:1}$	
	OD_{600}	wet cell pellet (g)	OD_{600}	wet cell pellet (g)
wild type	24,7	1,1	17,4	1,3
G1250S	21,9	1,1	19,2	1,4
G1250S M1251W	15,6	1,0	19,5	1,5
I306A	23,1	1,1	19,2	1,4
I306A G1250S M1251W	15,9	1,1	18,8	1,4
R1834K G1250S M1251W	3,5	0,2	22,0	1,5
I306A R1834K G1250S M1251W	5,4	0,5	20,4	1,7
R1834K	20,7	1,1	24,1	1,4
I306 R1834K	22,5	1,2	20,3	1,5
I306A G1250S	15,6	1,3	18,9	1,4
R1834K G1250S	10,2	0,9	19,6	1,4
I306A R1834K G1250S	14,0	1,1	18,3	1,3
G1250S F1279Y	0,1	0,3	17,5	1,4
I306A G1250S F1279Y	0,3	0,3	16,7	1,3
R1834K G1250S F1279Y	0,1	0,3	15,8	1,4
I306A R1834K G1250S F1279Y	0,1	0,3	17,0	1,5
I306A F1279Y	15,6	1,2	16,9	1,3
I306A G1250S M1251W F1279Y	- *	- *	18,2	1,4
I306A F1279W	- *	- *	17,7	1,3
I306A G1250S F1279W	- *	- *	21,8	1,4
I306A G1250S M1251W F1279W	- *	- *	18,4	1,3
I306A R1834K F1279Y	4,3	0,6	21,1	1,4
I306A R1834K G1250S M1251W F1279Y	- *	- *	20,5	1,3
I306A R1834K F1279W	- *	- *	23,6	1,4
I306A R1834K G1250S F1279W	- *	- *	15,8	1,4
I306A R1834K G1250S M1251W F1279W	- *	- *	21,1	1,3

Supplementary Table 4: Primers for site directed mutagenesis. The mutation site is indicated in bold typing, while the overlap between the primers is underlined.

I306A for	TTCTTC**GCT**GGTGTTCGTTGTTACGAAGCATACCCAAACACTTCC
I306A rev	ACACC**AGC**G AAG AATAATACAGTAATTGCTTTTCTTACGGAGACG
R1834K for	AGTTGTGTTCTAC**AAA**GGTATGACTATGCAAGTTGCTGTTCC
R1834K rev	CATAGTCATACC**TTT**GTAGAACACAACTTCAACTAAAGATTCGATAGAC
G1250S for	TCTGGTTCT**TCT**ATGGGTGGTGTTTCTGCCTTACG
G1250S rev	CAT**AGA**AGAACCAGAACAGTTACCAACCTCAGAAACATGTACG
M1251W for	TCTGGTTCT**TCTTGG**GGTGGTGTTTCTGCCTTACG
M1251W rev	**CCAAGA**AGAACCAGAACAGTTACCAACCTCAGAAACATGTACG
F1279Y for	ATTTTACAAGAATCA **TAT** ATCAACACCATGTCCGC
F1279Y rev	TGATTCTTGTAAAATATCATTTTGGACAGGC
pADH2-Fas1 (*[1])	GAACCGTGAGATAGGGTTAATGGTCTTGTGGAGTAAGCGTCCATTGTGTATTACGATAT AGTTAATAGTTGATAG
pADH2-Fas2 (*[2])	GCAAAATATGAGCTAATTCTTGCTCAACTTCCGGCTTCATTGTGTATTACGATATAGTT AATAG
pRS-ADH2 (*[3]) (FAS1 and FAS2)	CAGTGAATTGTAATACGACTCACTATAGGGCGAATTGGAGCTGGCAAAACGTAGGG GCAAACAAACG

*[1]amplification of ADH2 promoter, overhang homologous to FAS1 gene in pRS315
*[2]amplification of ADH2 promoter, overhang homologous to FAS2 gene in pRS313
*[3]amplification of ADH2 promoter, overhang homologous to vector pRS315 and pRS313
